# Cerebral critical closing pressure and resistance-area product: the influence of dynamic cerebral autoregulation, age and sex

**DOI:** 10.1177/0271678X211004131

**Published:** 2021-04-04

**Authors:** Ronney B Panerai, Victoria J Haunton, Osian Llwyd, Jatinder S Minhas, Emmanuel Katsogridakis, Angela SM Salinet, Paola Maggio, Thompson G Robinson

**Affiliations:** 1 Cerebral Haemodynamics in Ageing and Stroke Medicine (CHiASM) Research Group, Department of Cardiovascular Sciences, University of Leicester, Leicester, UK; 2 NIHR Leicester Biomedical Research Centre, British Heart Foundation Cardiovascular Research Centre, Glenfield Hospital, Leicester, UK; 3 Department of Vascular Surgery, Wythenshawe Hospital, Manchester Foundation Trust, Manchester, UK; 4 Neurology Department, Hospital das Clinicas, School of Medicine, University of Sao Paulo, Sao Paulo, Brazil; 5 Neurology Department, ASST Bergamo EST (BG), Italy

**Keywords:** Cerebral blood flow, transfer function analysis, myogenic mechanism, metabolic regulation, autoregulation index

## Abstract

Instantaneous arterial pressure-flow (or velocity) relationships indicate the existence of a cerebral critical closing pressure (CrCP), with the slope of the relationship expressed by the resistance-area product (RAP). In 194 healthy subjects (20–82 years, 90 female), cerebral blood flow velocity (CBFV, transcranial Doppler), arterial blood pressure (BP, Finapres) and end-tidal CO_2_ (EtCO_2_, capnography) were measured continuously for five minutes during spontaneous fluctuations of BP at rest. The dynamic cerebral autoregulation (CA) index (ARI) was extracted with transfer function analysis from the CBFV step response to the BP input and step responses were also obtained for the BP-CrCP and BP-RAP relationships. ARI was shown to decrease with age at a rate of −0.025 units/year in men (p = 0.022), but not in women (p = 0.40). The temporal patterns of the BP-CBFV, BP-CrCP and BP-RAP step responses were strongly influenced by the ARI (p < 0.0001), but not by sex. Age was also a significant determinant of the peak of the CBFV step response and the tail of the RAP response. Whilst the RAP step response pattern is consistent with a myogenic mechanism controlling dynamic CA, further work is needed to explore the potential association of the CrCP step response with the flow-mediated component of autoregulation.

## Introduction

Assessment of dynamic cerebral autoregulation (CA) in humans is usually performed by expressing the transient response of cerebral blood flow (CBF) to rapid changes in arterial blood pressure (BP), based on unidimensional metrics, such as the rate of regulation (RoR),^1^ the autoregulation index (ARI),^2^ or the mean flow index (Mx).^3^ Although single indices based on pre-established thresholds of abnormality facilitate the measurement of changes in dynamic CA performance in physiological studies, and the identification of patients with impaired CA,^4–6^ it is unlikely that unidimensional scales can capture the complexity of the multiple mechanisms contributing to the dynamic response, involving myogenic, metabolic and neurogenic pathways.^7–10^ Transfer function analysis (TFA) is one approach that can provide multiple indices (three distinct frequency bands for each of gain, phase and coherence parameters),^11^ but these are often applied in isolation.^12^

**Figure 1.**
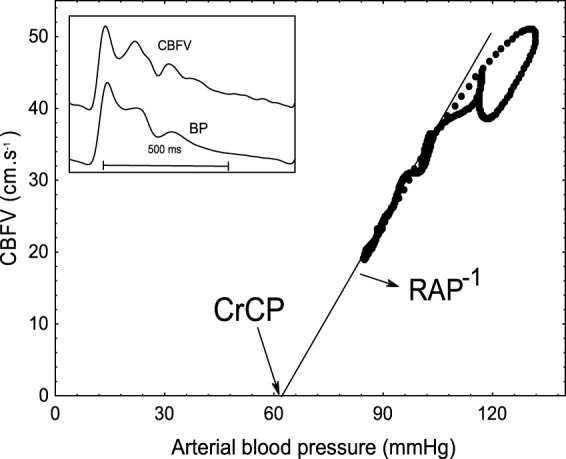
Scatter diagram of cerebral blood flow velocity (CBFV, right MCA) as a function of arterial blood pressure (BP), for a single cardiac cycle from a healthy 48 year-old male subject. The inset shows the corresponding waveforms of CBFV and BP. Critical closing pressure (CrCP) is determined by the intersection of the best-fit line with the BP axis. RAP is given by the inverse slope of the best-fit line. In this subject, CrCP is much higher than the population average (Table 1).

Ideally, comprehensive metrics of dynamic CA should also provide greater insight of underlying physiological processes, instead of empirical indices that simply reflect the rate of change of the CBF response (e.g. RoR, ARI), or its correlation with changes in cerebral perfusion pressure (e.g. Mx). One attractive possibility is to express the CA response based on the vasomotor properties of small arteries and arterioles, that ultimately result in changes in cerebrovascular resistance (CVR). To this end, it has been shown that a positive step change in mean arterial BP (MAP) leads to a gradual increase in CVR, reaching a plateau after approximately 5 s.^13–15^ One major limitation of this approach is that, by defining CVR as the ratio of MAP to mean CBF (or CBF velocity as usually measured with transcranial Doppler [TCD]), the presence of a critical closing pressure (CrCP) in the cerebral circulation^16^ is not taken into account. The CrCP cannot be identified using mean beat-to-beat values of CBF and BP as it requires techniques, with a high temporal resolution, that can provide a continuous measurement of CBF, such as TCD, where the instantaneous relationship between CBFV and BP can be expressed for each cardiac cycle. Using the scatter diagram of CBFV versus BP, it is then possible to determine the CrCP for each cardiac cycle, as the BP value where CBFV would reach zero, by extrapolation of the linear relationship, as exemplified in Figure 1. In his seminal communication, Burton^17^ interpreted CrCP of the pulmonary circulation as resulting from the balance between transmural pressure and wall tension, thus including the contribution of vascular smooth muscle activity. In the cerebral circulation, the existence of a significant CrCP has been confirmed by multiple studies.^16,18,19^

To take into account the contribution of CrCP, a scatter diagram between CBFV and BP has been modelled for each cardiac cycle as a linear relationship,^16^ where CrCP represents the intercept with the horizontal axis (that is CBFV = 0) and the slope of the straight line represents the inverse of the resistance-area product (RAP, Figure 1).^20^ RAP is a more rigorous expression of the vascular resistance index derived with TCD, considering that absolute flow is the product of cross-sectional area by the mean of the blood velocity profile across the vessel diameter. In a previous study, we have demonstrated that the dynamic responses of both RAP and CrCP to a sudden elevation in BP, known as a positive step change, and often referred to as the ‘step response’, can be derived from spontaneous fluctuations in BP, with CrCP step response being more sensitive to changes in PaCO_2_ than the corresponding step response for RAP.^21^ This novel approach allows for a more in-depth analysis of dynamic CA using metrics that are not only multidimensional, but also much closer to the physiological mechanisms effecting the CA response.

Hypercapnia is often used as a surrogate of dynamic CA impairment,^21–23^ but it is not clear to what extent it can mimic impairment of the myogenic response, or if its effect is mediated by impacting mainly on metabolic pathways.^24,25^ We have previously described the effects of hypercapnia on the CrCP and RAP step responses,^26^ but in this study we aimed to understand how these responses are affected by dynamic CA, as reflected by ARI. ARI has been validated as a sensitive index of dynamic CA, with extensive literature describing its use in physiological and clinical studies.^2,12,27^ In summary, to better understand the dependence of RAP and CrCP step responses on phenotypical characteristics, we studied a large population of healthy subjects to test the hypotheses that these step responses are influenced by age, sex and the effectiveness of dynamic CA. The importance of this study, therefore, is to expand the amount of information that can be extracted from continuous measurements of BP and CBFV in order to improve our understanding and assessment of dynamic CA in humans.

## Methods

### Ethical approval

All studies were approved by UK Research Ethics Committees from Northampton (11/EM/0369), Southampton and South West Hampshire (10/H0502/1), North East-Newcastle & North Tyneside (14/NE/1003) and the University of Leicester (jm591-c033). All procedures were conducted in accordance with the Declaration of Helsinki and all participants provided written informed consent.

### Subjects and measurements

Healthy subjects, 18 years of age or older, without any history or symptoms of cardiovascular, neurological or respiratory disease, were studied with a standard protocol for recordings at rest and included in the Leicester database.^6,22,24,28–30^

Volunteers were asked to avoid heavy exercise, caffeine, alcohol and nicotine for at least 4 h before attending the University of Leicester’s Cerebral Haemodynamics in Ageing and Stroke Medicine (CHIASM) research laboratory, where measurements were performed with minimal auditory or visual distraction. BP was recorded continuously using a Finapres/Finometer device (FMS, Finapres Measurement Systems, Arnhem, Netherlands), attached to the middle finger of the left hand. Heart rate was derived from a 3-lead electrocardiogram (ECG). End-tidal CO_2_ (EtCO_2_) was measured continuously via nasal prongs (Salter Labs) with a capnograph (Capnocheck Plus). Bilateral CBFV was recorded with transcranial Doppler ultrasound (TCD, Viasys Companion III; Viasys Healthcare, PA, USA) from the middle cerebral arteries (MCAs) using 2 MHz probes secured in place with a head-frame. Systolic and diastolic BP were measured by standard brachial sphygmomanometry (OMRON 705IT) before each 5 min recording. The servo-correcting mechanism of the Finapres/Finometer was switched on and then off prior to measurements.

Data were continuously recorded onto a data acquisition system (PHYSIDAS, Department of Medical Physics, University Hospitals of Leicester) for subsequent off-line analysis using a sampling rate of 500 samples/s.

### Experimental protocol

In all cases, data extracted from the Leicester database, corresponded to the first 5 min recording performed in different studies, with subjects breathing normally at rest in the supine position, with the head elevated at 30°. All recordings were performed by investigators trained to rigorous standard procedures.

### Data analysis

All signals were visually inspected to identify artefacts; noise and narrow spikes (<0.1 s) were removed by linear interpolation. CBFV channels were subjected to a median filter and all signals were low-pass filtered with a 8th order Butterworth filter with cut-off frequency of 20 Hz. BP was calibrated at the start of each recording using systolic and diastolic values obtained with sphygmomanometry, using our data editing software written in FORTRAN. The R–R interval was then automatically marked from the ECG and beat-to-beat heart rate (HR) was plotted against time. Occasional missed marks caused spikes in the HR signal; these were manually removed by remarking the R–R intervals for the time points at which they occurred. CrCP and RAP were calculated using the 1st harmonic method.^15^ For each cardiac cycle (Figure 1), the first harmonic (fundamental frequency) of the BP (P_1_) and CBFV (V_1_) waveforms were extracted and RAP was calculated by the ratio P_1_/V_1_. The corresponding CrCP for the same cardiac cycle is then given by CrCP = P_0_ − RAP.V_0_, where P_0_ and V_0_ are the corresponding mean values of BP and CBFV for the cardiac cycle. The end of each expiratory phase was detected in the EtCO_2_ signal, linearly interpolated, and resampled with each cardiac cycle. Mean, systolic and diastolic BP and CBFV values were calculated for each cardiac cycle. Beat-to-beat data were spline interpolated and resampled at 5 samples/s to produce signals with a uniform time-base.

#### Sub-component analysis

Assuming that the instantaneous BP-CBFV relationship can be expressed by a linear model, leads to CBFV=(BP − CrCP)/RAP for each cardiac cycle.^16^ For small changes in RAP, it is possible to express this relationship as:^26,31^

(1) $$V_{\mathrm{MCA}}=V_{\mathrm{MAP}}+V_{\mathrm{CrCP}}+V_{\mathrm{RAP}}$$

Where V_MCA_ is the percent change in CBFV during spontaneous fluctuations in MAP and V_MAP_, V_CrCP_ and V_RAP_ are the corresponding sub-components, expressing their individual percent contribution to explain the overall changes in V_MCA._^26,31^

TFA of the V_MAP_-V_CBFV_ relationship was performed using Welch’s method ^32^ with data segmented with 102.4 s duration and 50% superposition.^11^ Mean values of V_MAP_ and V_CBFV_ were removed from each segment and a cosine window was applied to minimise spectral leakage. The squared coherence function, gain and phase frequency responses were calculated from the smoothed auto- and cross-spectra using standard procedures.^11,33^ The V_CBFV_ step response to the V_MAP_ input was estimated using the inverse fast Fourier transform of gain and phase.^27,34^ ARI was extracted by using the normalised minimum square error (NMSE) fit between the CBFV step response and one of the 10 model ARI curves proposed by Tiecks et al.^2^ ARI values were only accepted if the mean squared coherence function for the 0.15–0.25 Hz frequency interval was above its 95% confidence limit, adjusted for the corresponding degrees of freedom, and the NMSE was ≤0.30.^35^ Similar to the V_MAP_-V_CBFV_ dynamic relationship, TFA was also performed for the V_MAP_-V_RAP_ and V_MAP_-V_CrCP_ relationships, and corresponding step responses were obtained with the inverse FFT.^26^ A separate analysis of the coherence function was performed for these two transfer functions and step responses were only accepted if the mean value of coherence over a *Δf* = 0.1 Hz frequency interval was above the 95% confidence limit of 0.189 as this value is only dependent on *Δf* and not on its position on the frequency spectrum.^35,36^ The frequency interval where coherence was largest for the V_MAP_–V_RAP_ and V_MAP_–V_CrCP_ transfer functions was chosen in each case from the results presented below.

Based on the linear properties of the FFT, a similar relationship between the responses to a step change in MAP can be written as the summation in equation (1).^26^ By changing the order of the parcels, it is possible to express the step change in MAP as:

(2) $${\mathrm{SRV}}_{\mathrm{MAP}}^{*}={\mathrm{SRV}}_{\mathrm{MCA}}-{\mathrm{SRV}}_{\mathrm{CrCP}}-{\mathrm{SRV}}_{\mathrm{RAP}}$$

where SRV*_MAP_ is an estimate of the MAP step change, based on the summation of the three other step responses (SR). Therefore, the expression above works as a ‘checksum’ for the correctness of the three distinct SR estimated for V_MCA_, V_CrCP_ and V_RAP_.

### Statistical analysis

SRV_MCA_ was only accepted based on the dual criteria of coherence above the 95% confidence limit and a NMSE ≤0.30 for fitting of the Tiecks model.^2,35^ SRV_CrCP_ and SRV_RAP_ were only accepted if their corresponding transfer functions both had mean coherence above the 95% confidence limit, for the frequency interval described below, for at least one of the hemispheres, assuming that SRV_MCA_, and the corresponding value of ARI, had also been accepted. Specifically, for the right or left hemisphere to be accepted, all three step responses for that hemisphere had to be accepted. SRs and ARI values were averaged for the right and left hemispheres when both were accepted.

Data distribution was tested with the Shapiro-Wilk W statistic. With the exception of the mean coherence, all other parameters were normally distributed.^6^ The distribution of coherence was expressed by its median [interquartile limits], all other parameters as mean ± SD. Differences between parameters were assessed using the paired Student’s *t*-test or the Wilcoxon test. In each of the SR, values were averaged for three distinct time intervals; T1 (0.6–1.4 s), T2 (3–4 s) and T3 (7–10 s). T1 corresponds to the peak of the SRV_MCA_, T2 the beginning of its plateau phase, and T3 the tail of the response (Figures 3(a) and 5(a)).

For each of the three time intervals (T1-T3), the General Linear Model (GLM) and multivariate linear regression were adopted to describe the dependence of SRV_CBFV_, SRV_RAP_ and SRV_CrCP_ on ARI and the effect of sex and age as co-variates. A *p-*value of <0.05 indicated statistical significance.

## Results

A total of 236 subjects met the selection criteria.

### Transfer function analysis coherence

**Figure 2.**
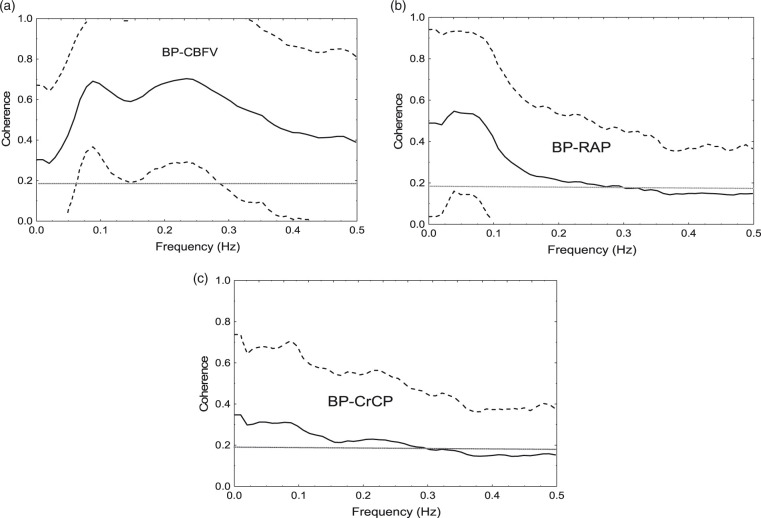
Population average coherence (solid line) for 474 hemispheres for the transfer function between MAP (input) and CBFV (a), RAP (b) and CrCP (c) as output. The dashed line represents the 90% confidence interval, limited to the coherence range 0–1. The horizontal dotted line marks the 95% confidence limit for coherence, based on the mean coherence for a frequency interval *Δf* = 0.1 Hz.

Mean and 90% confidence intervals of the squared coherence function for the corresponding 472 hemispheres are presented in Figure 2 for the transfer functions between V_MAP_ and V_CBFV_, V_RAP_, and V_CrCP_, respectively. For the V_MAP_ − V_RAP_ and V_MAP_ − V_CrCP_ transfer functions, the largest values of coherence were obtained in the 0–0.1 Hz frequency interval, whilst for the V_MAP_ − V_CBFV_ transfer function, the highest values of coherence, above the range where dynamic CA shows non-linear behaviour, was in the interval 0.15–0.25 Hz. Hemispheres with mean coherence values below the 95% confidence limit were not accepted, leading to the rejection of 36 subjects, mainly due to low coherence in the V_MAP_-V_CrCP_ transfer function (Figure 2(c)). In addition, six further subjects were rejected due to NMSE values above 0.3 for fitting the Tiecks model to the V_CBFV_ step response (see Methods). The resulting 194 subjects (104 males) had ages ranging from 20 to 82 years old (mean 51.7 ± 15.2).

### Influence of age and sex

None of the bilateral parameters in Table 1 showed interhemispheric differences, but CBFV was higher in females compared to males, whilst systolic BP and RAP were higher in men in relation to women. Individual values of EtCO_2_ and CBFV, averaged over the 5 min recording, did not show a significant correlation. As discussed below, due to this lack of association, EtCO_2_ was not included in any further analyses. On the other hand, individual values of ARI were significantly associated with age in male subjects, but not in females (R = 0.0891, p = 0.40). In males (n = 104), correlation was R = 0.224 (p = 0.02), and the predicted linear equation was ARI = 7.05–0.0251*AGE. Apart from its influence on the relationship between ARI and age, sex was not a significant co-factor in any other analyses based on GLM or multivariate regression.

**Table 1.** Subject characteristics and physiological parameters according to sex.

Parameter	Female (n = 90)	Male (n = 104)	p-value
Age (years)	51.1 ± 14.9	52.3 ± 15.5	0.57
ARI	5.72 ± 1.57	5.73 ± 1.74	0.31
CBFV_R_ (cm.s^−1^)	56.6 ± 13.7	52.2 ± 12.8	0.023
CBFV_L_ (cm.s^−1^)	57.2 ± 11.9	51.0 ± 10.2	0.0002
Systolic BP (mmHg)	124.1 ± 19.7	133.4 ± 20.3	0.002
Mean BP (mmHg)	88.5 ± 11.3	90.9 ± 10.6	0.14
Diastolic BP (mmHg)	70.2 ± 9.9	71.7 ± 10.7	0.31
EtCO_2_ (mmHg)	38.75 ± 2.66	39.28 ± 3.35	0.25
Heart rate (bpm)	66.5 ± 9.9	63.9 ± 8.3	0.060
CrCP_R_ (mmHg)	29.5 ± 15.3	29.5 ± 14.0	0.98
CrCP_L_ (mmHg)	30.7 ± 13.7	29.2 ± 14.0	0.49
RAP_R_ (mmHg.s.cm^-1^)	1.12 ± 0.43	1.28 ± 0.44	0.013
RAP_L_ (mmHg.s.cm^-1^)	1.09 ± 0.38	1.32 ± 0.45	0.0002

Note: Physiological parameters were averaged for 5-min duration of recordings.

p-values from independent t-test for differences between males and females.

ARI: autoregulation index; CBFV: cerebral blood flow velocity; EtCO_2_: end-tidal CO_2_; CrCP: critical closing pressure; RAP: resistance area-product; R/L: right/left hemisphere.

### CBFV, CrCP and RAP step responses

**Figure 3.**
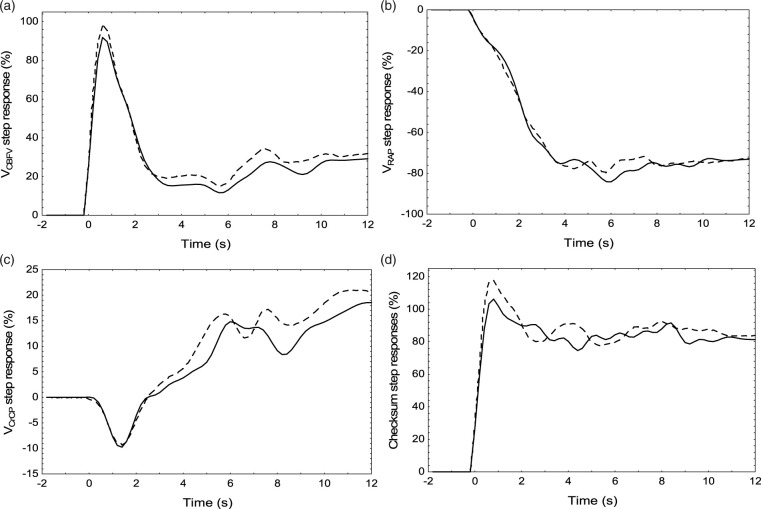
Representative step responses for V_CBFV_ (a), V_RAP_ (b) and V_CrCP_ (c) for the right (continuous line) and left (dashed line) MCAs from a 75 year-old male subject. The mean ARI for the two hemispheres was 6.03, very close to the population median ARI = 6.02. (d) Checksum of the a–c step responses, reflecting the cumulative numerical estimation errors.

The step response for V_CBFV_ (Figure 3(a)) showed the characteristic rapid rise at t = 0, followed by a more gradual return to baseline, according to an ARI value of 6.03 for this subject. SRV_RAP_ (Figure 3(b)) had a gradual, exponential-like reduction, reaching a plateau after 4 s. On the other hand, SRV_CrCP_ (Figure 3(c)) presented a more complex temporal pattern, with a small trough followed by a slow continuous rise. The ‘checksum’ of the step responses (Figure 3(d)) confirms the validity of equation (2) (Methods). Its departure from a perfect step function provides a measure of the combined numerical errors of estimation of the three distinct step responses. Following the peak in the V_CBFV_ step response at t = 1 s, its subsequent reduction towards the original baseline was caused by the drop in SRV_RAP_, but, with the corresponding gradual rise in SRV_CrCP_, the tail end of the V_CBFV_ step response remained around values of approximately 25% (Figure 3(a)).

**Figure 4.**
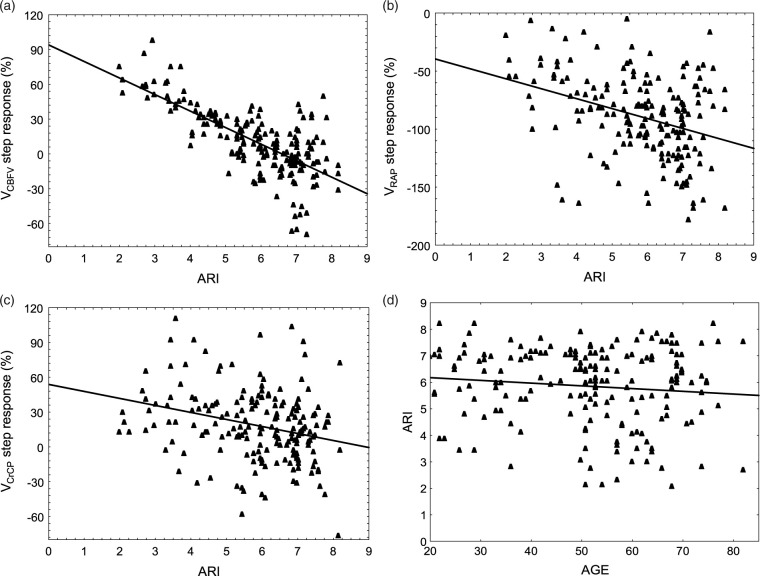
Averaged step response values for the time interval T3 (7–10 s) for V_CBFV_ (a), V_RAP_ (b) and V_CrCP_(c) as a function of the ARI (n = 194). The straight line is the best linear fit with p < 0.0001 in all three cases. (d) Linear association between ARI and age (r = −0.159, p = 0.021).

With the exception of SRV_RAP_ during interval T1, ARI had a highly significant influence on the three step responses (Table 2), whilst age was only significant for SRV_CBFV_ during time interval T1, and SRV_RAP_ during T3 (Table 2). During T3, the three step responses were strongly associated with ARI (Table 2 and Figure 4). The large correlation coefficient between SRV_CBFV_ (Figure 4(a)) and the ARI (r = 0.76) was to be expected from the Tiecks model,^2^ but the two other associations (Figure 4(b) and (c)) have not been reported previously.

**Table 2.** Multiple linear regression modelling of V_CBFV_, V_RAP_ and V_CrCP_ responses to a step change in MAP.

Time interval	Regression model (%)	R	p-value ARI	p-value Age
T1	SRV^1^_CBFV_ = 105.64–3.78*ARI – 0.182*Age	0.353	<0.0001	0.027
	SRV^1^_RAP_: NS	0.065	NS	NS
	SRV^1^_CrCP_ = 30.65–5.17*ARI	0.285	<0.0001	NS
T2	SRV^2^_CBFV_ = 84.78–11.99*ARI	0.848	<0.0001	NS
	SRV^2^_RAP_ = −22.46–7.41*ARI	0.467	<0.0001	NS
	SRV^2^_CrCP_ = 28.45–4.35*ARI	0.281	<0.0001	NS
T3	SRV^3^_CBFV_ = 101.98–15.52*ARI	0.763	<0.0001	NS
	SRV^3^_RAP_ = −43.57–10.59*ARI + 0.326*Age	0.491	<0.0001	0.047
	SRV^3^_CrCP_ = 47.64–5.04*ARI	0.267	<0.0001	NS

T1-T3: time intervals from step responses corresponding to 0.6–1.4 s, 3–4 s and 7–10 s, respectively; SRV^i^_CBFV_: mean values of V_CBFV_ step response for time intervals T_i_; SRV^i^_RAP_: mean values of V_RAP_ step response for time intervals T_i_; SRV^i^_CrCP_: mean values of V_CrCP_ step response for time intervals T_i_; R: correlation coefficient; p-value ARI: p-value for ARI slope coefficient from multivariate regression analysis; p-value age: p-value for the age slope coefficient from multivariate regression analysis.

**Figure 5.**
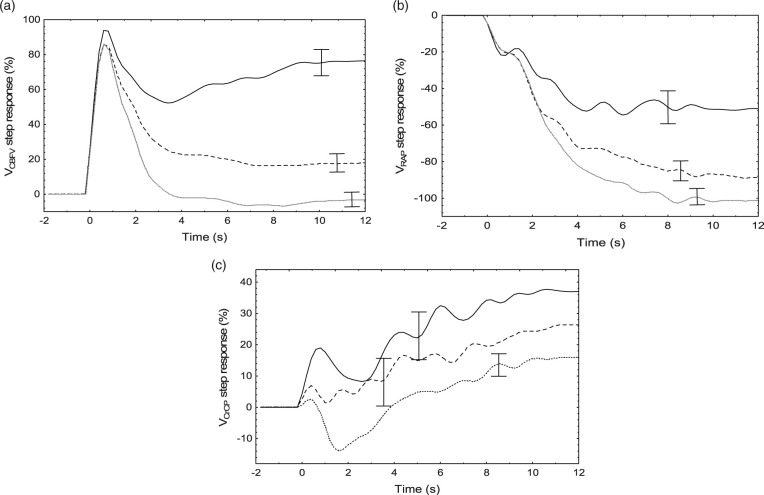
Population mean step responses for V_CBFV_ (a), V_RAP_ (b) and V_CrCP_ (c) for ARI values in the ranges 0–4 (n = 29, continuous line), 4–6 (n = 65, dashed line) and 6–9 (n = 100, dotted line). The error bars represent the largest ±1SE at the point of occurrence.

For different ranges of ARI values, there were distinct temporal patterns of the step responses (Figure 5). With increases in ARI, the tail end (7–10 s) of the V_RAP_ and V_CrCP_ step responses were both shifted down (Table 2, Figure 5(b) and (c)). On the other hand, the step response for V_CrCP_ (Figure 5(c)) also showed changes in its temporal pattern around T1 (0.6–1.4 s), as confirmed by the significant regression in Table 2.

## Discussion

### Main findings

Using a relatively large number of recordings from healthy subjects, covering a wide age range, we combined TFA with sub-component analysis to describe the breakdown of the CBFV dynamic response to a step change in MAP, into two distinct step responses, namely SRV_RAP_ and SRV_CrCP_, and their dependence on the efficiency of CA, as well as on age and sex. The novelty of this approach, and the relevance of our findings, shows considerable potential for the use of SRV_RAP_ and SRV_CrCP_ to obtain a more in-depth understanding of dynamic CA in humans, refining our current abilities to identify deterioration of CA in different cerebrovascular conditions.

Given the conflicting reports in the literature about the influence of ageing on dynamic CA, the finding that ARI decreases with age in men, but not women, is particularly pertinent. Despite its influence on the relationship between age and ARI, sex was not found to affect the highly significant relationships between step response markers and ARI. The latter demonstrated how the well-known changes in the temporal pattern of SRV_CBFV_, reflecting changes in dynamic CA efficiency, are explained by corresponding changes in SRV_RAP_ and SRV_CrCP_. These findings, and their contribution to break the mould of unidimensional empirical indices can be regarded as a paradigm shift in our methods to quantify dynamic CA, with measures that are closer to the underlying physiological mechanisms responsible for CBF regulation. Moreover, we have also described the population distribution of the coherence function for the V_MAP_ − V_RAP_ and V_MAP_ − V_CrCP_ transfer functions, and the relevant finding that, in contrast to the V_MAP_ − V_CBFV_ transfer function, the former have maximum values in the range 0–0.1 Hz, as opposed to the 0.15–0.25 Hz observed for the latter. The implications of these findings are discussed in more detail below.

### Influence of age on dynamic CA

Although the main aim of our study was not to assess the effects of ageing on dynamic CA, the finding of a significant association between ARI and age is highly relevant, given the dominant view in the literature that ageing does not affect CA.^9,37–43^ Importantly though, age has been found to have an effect on CA in some studies.^42,44,45^ It is possible that other studies have missed the small effect that we found, corresponding to ΔARI = −0.0251/year (in men only), due to an interaction of multiple factors such as inter- and intra-subject variability, statistical power, the contribution of sex, and measurement protocols.^46^ The interplay between intra- and inter-subject variability of ARI can explain the difficulty of identifying the effect of age on CA, mainly when relatively small, mixed-sex cohorts are involved. In a group of healthy subjects with mean age 37.5 years, Brodie et al observed an intra-subject standard error of the mean (SEM) ARI of approximately 0.75 ARI units, which would translate to an ageing equivalent effect of 0.75/0.0251 = 29.9 years, according to our linear regression coefficient.^47^ On the other hand, when performing a longitudinal study over a period of 10 years, the same authors ^45^ identified a reduction in ARI of 1.1 units, considerably more than the 0.251 units predicted by the linear model in Table 2. The corollary of these studies is that the relatively high intra- and inter-subject variability of ARI, and other metrics of CA,^48^ can obfuscate the influence of age on CA. However, the subtle effects of age that we identified could become easier to detect with larger sample sizes. In a group of 544 individuals with mean age approximately 78 years, Deegan et al have shown that older women had ARI values that were higher than older men, by an average ΔARI = 0.91.^49^ Although a younger group was not included, their findings suggest agreement with our results since we identified an effect of ageing on CA of men, but not of women, who could then maintain the same CA efficiency throughout their life span, whilst men gradually deteriorated. In their study, Deegan et al^49^ estimated ARI values with the sit-to-stand protocol, that tends to induce changes in MAP substantially higher than those usually observed with spontaneous fluctuations in BP. The influence of larger oscillations in MAP to improve the reliability of CA metrics,^28,50^ and facilitate the identification of the effects of ageing, is also illustrated by the findings of Batterham et al, who recently reported reduced ARI values in older subjects performing the squat-stand manoeuvre.^44^ In both their younger and older groups, the sex ratio was 50%, but the influence of sex on the effect of age was not examined. Similarly, Smirl et al^42^ compared groups of older and younger subjects undergoing mild exercise combined with oscillatory lower body negative pressure, which induced relatively large changes in MAP at fixed frequencies of 0.05 and 0.10 Hz. In the older group, significant increases in TFA gain and reduced values of phase suggested that ageing reduces CA efficiency. Noteworthy, only male subjects were recruited.

In addition to its effects through ARI, age also affected the step response patterns of SRV_CBFV_ during T1, and SRV_RAP_ during T3 (Table 2). However, in both cases, the influence of age was relatively small, when compared to the contribution of ARI in the linear regression equations (Table 2). Importantly, in neither case was there a significant effect of sex, suggesting that the underlying mechanisms might be distinct from those mediating the effect of ageing on ARI. During T1, age reduced the upsurge of the SRV_CBFV_ peak. Speculatively, this could reflect increased narrowing of cerebral vessels with ageing. In the case of SRV_RAP_, the significance of age was borderline (p = 0.047, Table 2) and the overall effect relatively small. For each yearly increase in age, the corresponding difference in SRV_RAP_, would be equivalent to a reduction in ARI of 0.18 units.

Further work is needed to fully understand the effects of ageing on CA, taking into consideration the interaction with sex, ideally using a protocol that induces large oscillations in MAP in a sufficiently large cohort of individuals, to allow reliable statistical modelling. Hypertension, and other risk factors, that can influence the structure and adaptation of cerebral vessels, should also be carefully controlled for to identify sub-groups of individuals where ageing might have a more detrimental effect on CA and other mechanisms of CBF regulation.^46^ Moreover, it is also possible that ageing is only a reflection of the key physiological mechanisms behind alterations in ARI and that other variables, such as arterial stiffness or endothelial dysfunction, might provide more significant correlations with ARI.

### Mechanisms of CBF regulation

In humans, assessment of CA using either the static or the dynamic approach, has always been performed with ‘black-box’ models, adopting TFA or a number of other metrics.^5,12,51,52^ These indices have allowed identification of CA impairment in a wide range of cerebrovascular conditions.^12^ However, in human physiological studies, unidimensional scales have been frustratingly poor in allowing us to understand the way CA interacts with other cardiovascular regulatory mechanisms, for example the baroreflex, or physiological interventions, such as exercise, posture, or temperature changes, when compared to the wealth of information that can be derived from animal studies. The possibility of increasing the amount of information that could be extracted in human studies of CA, by incorporating the dynamic response of RAP and CrCP to rapid changes in MAP, represents a small, but highly relevant step towards our ability to refine current methodological approaches. RAP and CrCP not only increase the dimensionality of CA assessment, but do so in a way that could be of physiological relevance. Several studies have suggested that RAP may more sensitively reflect the myogenic response, while CrCP would be influenced mainly by metabolic mechanisms.^31,53^ Although hypercapnia induces significant increases in CBFV and depression of CA, the contribution of RAP to explain these changes has been minimal or entirely absent.^23,26,54^ Moreover, time-varying changes in RAP have been closely associated with a response to preceding changes in MAP, and not with EtCO_2_^25^^,.^^25,31,55,56^ On the other hand, different authors have reported a strong association between CrCP and PaCO_2_^16,23^ and dynamic analyses have also shown that CrCP could explain better the changes in CBFV resulting from neural stimulation than RAP.^31,54,57^ In a recent study, SRV_CrCP_, but not SRV_RAP_, explained the corresponding changes in SRV_CBFV_ in response to hypercapnia.^26^

In this study, estimates of SRV_RAP_ (Figures 3(b) and 5(b)) were in broad agreement with previous estimates of RAP, or CVR response to a step change in MAP,^14,15,54,58^ remembering that in our case the SRV_RAP_ curves are inverted, to represent the corresponding reduction in V_CBFV_, following a rise in RAP. What has not been described previously though, is how the SRV_RAP_ temporal pattern varies with the efficiency of dynamic CA (Figure 5(b)) and the linear association of the SRV_RAP_ with ARI at T2 (Table 2), and both ARI and age at T3 (Table 2, Figure 5(b)). As shown in Figure 5(b), with a step change in MAP, the gradual rise in RAP leads to a reduction in SRV_RAP_, that becomes more negative as ARI increases. This reduction in SRV_RAP_ was present, even for reduced values of ARI (0-4), although the corresponding SRV_CBFV_ remained elevated (Figure 5(a)). To explain the elevated values of SRV_CBFV_, for low values of ARI, it is necessary to take into account the temporal pattern of SRV_CrCP_ as well (Figure 5(c)). With SRV_CrCP_ rising continuously, it compensates for the drop in SRV_RAP_, thus maintaining SRV_CBFV_ elevation. For ARI = 0, we could expect that SRV_CBFV_ would be a constant plateau,^2^ but this was not the case because the interval considered also included values as high as ARI = 4. With increasing values of ARI, the contribution of SRV_CrCP_ was gradually reduced (Figure 5(c)), and the corresponding drop in SRV_CBFV_, following a step change in MAP, was dominated mainly by corresponding changes in SRV_RAP_ (Figure 5(b)).

Temporal patterns of SRV_RAP_ (Figures 3(b) and 5(b)) are physiologically plausible, and are in agreement with previous reports mentioned above. On the other hand, SRV_CrCP_ has only been reported by our group, first in a NVC study,^54^ and more recently in the comparison between hypercapnia and normocapnia.^26^ Interpretation of its temporal pattern (Figures 3(c) and 5(c)) is much more challenging than for SRV_RAP_. To start with, why has SRV_CrCP_ shown its largest changes for the lowest range of ARI (0–4) in Figure 5(c)? To address this question, it is important to notice that the end values of the SRV_CrCP_ curves are varying inversely with the corresponding values of SRV_CBFV_ (Figure 5 (a)). One interpretation that would fit with this observation is that SRV_CrCP_ reflects the vasodilation resulting from wall shear stress, a mechanism posited to depend on endothelial release of nitric oxide (NO) and other mediators.^59,60^ This interpretation is consistent with our previous findings demonstrating that SRV_CrCP_ showed an even larger rise for t > 4 s during hypercapnia, when compared to normocapnia,^26^ although in that study we did not stratify SRV_CrCP_ by ARI. The contribution of flow-mediated control of vascular smooth muscle is well established in experimental studies of CA and NVC,^59^ but it is not an exaggeration to say that it has been largely ignored in the literature on human studies of CA. What is still controversial, in both *in vitro* and *in vivo* studies of flow-mediated vasomotor activity though, is the directional effects of increases in flow, that could lead to vasoconstriction, vasodilation, or a bi-phasic response.^59^ Our speculation, that SRV_CrCP_ could express the contribution of flow-mediated mechanisms to the CA response, would be dependent on vasodilation being the dominant effect of increases in flow in the vascular bed supplied by the MCA. However, in isolated human MCA arteries, Toth et al have shown that increases in flow lead to vasoconstriction.^61^ On the other hand, in a more intact preparation, using a cranial window in rats, Paravicini et al have observed a predominantly vasodilatory response.^60^ Much more work is needed to understand the anatomical and functional heterogeneity of flow-mediated mechanisms,^59^ but, in human studies, it would be particularly relevant to modulate endothelial function to observe its effects on SRV_CrCP_, for example with a bolus of N^G^-monomethyl-L-arginine (L-NMMA), an inhibitor of NO synthase, that was shown to affect dynamic CA.^62^ The temporal patterns of SRV_CrCP_ for t < 3 s are also intriguing, mainly when comparing the directional changes for very low (0-4) and very high (6-9) values of ARI (Figure 5(c)). Given that CrCP is highly dependent on active wall tension,^16,17,63^ it would be reasonable to speculate that the temporal increase in VR_CrCP_ with poor CA (low ARI) could be caused by vessel stretching due to lack of active wall tension, whilst the opposite would be observed with an active CA, where reflex smooth muscle contraction, leading to increased wall tension, would counteract the sudden change in intravascular pressure.

### Methodological considerations

CrCP and RAP are not directly measured quantities, but are derived from the instantaneous pressure-velocity relationship for each cardiac cycle.^16^ As such, these parameters are highly sensitive to noise and baseline fluctuations in the BP and CBFV signals, which in some cases can lead to non-physiological results, for example with negative values of CrCP.^64^ When obtaining estimates of CrCP and RAP for an entire recording, averaging values from a large number of cardiac cycle can mitigate the multiple sources of variability,^16^ but this is not feasible in estimates of SRV_CrCP_ and SRV_RAP_. Multiple approaches have been proposed to extract CrCP and RAP values from the pressure-velocity relationship.^15,64,65^ We have adopted the first harmonic method,^16^ based on its balanced performance for ‘static’ estimates of CrCP and dynamic estimates of SRV_RAP._^15^ Nevertheless, when combined with the TFA numerical procedures to estimate the three distinct step responses, the cumulative errors can be reflected in the SRV_MAP_, obtained as the ‘checksum’ in equation (1). Figure 3(d) shows one example, with a pronounced departure from a perfect step function, which is representative of the worst cases in our sample. From the perspective of incorporating estimates of SRV_CrCP_ and SRV_RAP_ in clinical and physiological studies, further work is needed to explore alternative algorithms that could improve the reliability of these estimates and their robustness to noise.

Further methodological advances would also be desirable in the modelling approach to convert the beat-to-beat time-series of CrCP and RAP into estimates of SRV_CrCP_ and SRV_RAP_. In this and previous work,^26^ we have adopted conventional TFA for this purpose and, despite their considerable inter-subject variability, the corresponding step responses were physiologically plausible (SRV_RAP_) or showed consistency in response to hypercapnia for different values of sex, age or ARI (SRV_CrCP_). One possibility that deserves further investigation is to obtain estimates of SRV_RAP_ and SRV_CrCP_ using time-domain models, such as we have demonstrated with autoregressive-moving average structures.^54^ An important feature of time-domain approaches is the possibility of performing multivariate analyses that could include co-variates, such as PaCO_2_, thus helping to clarify the physiological determinants of SRV_CrCP_ to allow further insight into its interpretation.

## Limitations

Some important limitations of the study have been addressed above. Other limitations include the assumption that the cross-sectional area of the MCA has remained constant during the 5 min recordings, to maintain a stable relationship between changes in CBFV and corresponding changes in CBF. This should be the case in our study given that large changes in MAP or EtCO_2_ were not present.^66,67^

Visual inspection of all raw data was part of our data analysis protocol, but slow drifts in the Finapres signal are difficult to detect^68^ and these could have caused distortions in estimates of SRV_CrCP_ and SRV_RAP_, although any larger distortions in MAP would have led to rejection of recordings due to our strict procedure for checking the statistical significance of the coherence function in the frequency interval 0–0.1 Hz. A similar consideration applies to the assumption that the spontaneous variability of BP with recordings at rest would be of sufficient magnitude to stimulate a dynamic CA response. Although the coherence criterion ascertained the reliability of estimates, it is possible that more robust estimates would have been obtained by removing recordings with reduced BP variability,^50^ or adopting an approach that induced larger changes in BP.^28,69^

Finally, despite our population comprising only healthy subjects, we have obtained values of ARI that were relatively low and would superpose with values that have been reported in clinical studies. Low values of ARI in healthy subjects have been reported in previous studies^6^ and their occurrence could be phenotypical, as it is often the case with many other physiological parameters, such as baroreceptor sensitivity, or due to methodological reasons.^70^ This is an area that requires further attention to improve the reliability of the ARI to detect pathological deterioration of CA.

## Conclusions

In a large number of healthy subjects, the dynamic CA index (ARI) decreased with age in men, but not in women. The dynamic responses of RAP and CrCP to a step change in MAP, estimated from spontaneous fluctuations in BP, were strongly influenced by ARI, but not by sex. For low values of ARI, suggestive of poor CA (ARI < 4), CrCP step response showed a marked contribution in association with elevated values of CBFV, but this effect was reduced for higher values of ARI when dynamic CA was mainly dominated by changes in RAP step response. Further physiological studies are needed to test the hypothesis that CrCP step response could express the contribution of flow-mediated mechanisms to CA response in humans.
